# Aberrations in Oxidative Stress Markers in Amyotrophic Lateral Sclerosis: A Systematic Review and Meta-Analysis

**DOI:** 10.1155/2019/1712323

**Published:** 2019-06-09

**Authors:** Zihao Wang, Zhile Bai, Xiaoyan Qin, Yong Cheng

**Affiliations:** Key Laboratory of Ethnomedicine for Ministry of Education, Center on Translational Neuroscience, College of Life and Environmental Sciences, Minzu University of China, Beijing 100081, China

## Abstract

Oxidative stress has been reported to be involved in the onset and development of amyotrophic lateral sclerosis (ALS). Data from clinical studies have highlighted increased peripheral blood oxidative stress markers in patients with ALS, but results are inconsistent. Therefore, we quantitatively pooled data on levels of blood oxidative stress markers in ALS patients from the literature using a meta-analytic technique. A systematic search was performed on PubMed and Web of Science, and we included studies analyzing blood oxidative stress marker levels in patients with ALS and normal controls. We included 41 studies with 4,588 ALS patients and 6,344 control subjects, and 15 oxidative stress marker levels were subjected to random-effects meta-analysis. The results demonstrated that malondialdehyde (Hedges' *g*, 1.168; 95% CI, 0.812 to 1.523; *P* < 0.001), 8-hydroxyguanosine (Hedges' *g*, 2.194; 95% CI, 0.554 to 3.835; *P* = 0.009), and Advanced Oxidation Protein Product (Hedges' *g*, 0.555; 95% CI, 0.317 to 0.792; *P* < 0.001) levels were significantly increased in patients with ALS when compared with control subjects. Uric acid (Hedges' *g*, -0.798; 95% CI, -1.117 to -0.479; *P* < 0.001) and glutathione (Hedges' *g*, -1.636; 95% CI, -3.020 to -0.252; *P* = 0.02) levels were significantly reduced in ALS patients. In contrast, blood Cu, superoxide dismutase, glutathione peroxidase, ceruloplasmin, triglycerides, total cholesterol, low-density lipoprotein, high-density lipoprotein, coenzyme-Q10, and transferrin levels were not significantly different between cases and controls. Taken together, our results showed significantly increased blood levels of 8-hydroxyguanosine, malondialdehyde, and Advanced Oxidation Protein Product and decreased glutathione and uric acid levels in the peripheral blood of ALS patients. This meta-analysis helps to clarify the oxidative stress marker profile in ALS patients, supporting the hypothesis that oxidative stress is a central component underpinning ALS pathogenesis.

## 1. Introduction

Amyotrophic lateral sclerosis (ALS) is a devastating neurodegenerative disease caused by cell death of both upper and lower motor neurons [1]. It is known that 90-95% of cases are sporadic and the remaining 5-10% of cases are due to genetic predisposition [2]. The onset of ALS usually begins at the age of 50 years old for genetically inherited cases and at the age of 60 years old for sporadic cases, although the disease can start at any age [2]. It is estimated that two to three people out of 100,000 are affected by ALS in the United States and Europe every year [3, 4], while the incidence of the disease in the rest of the world remains largely unknown. Due to the poorly understood etiology of ALS, there is no effective treatment for the disease, and most patients survive between 2 to 4 years after diagnosis [5]. FDA has approved two drugs (riluzole and edaravone) for the treatment of ALS, and riluzole is reported to increase the life expectancy of patients by 2 to 3 months [6]. Therefore, it is critical to elucidate the etiology of ALS to facilitate the development of novel therapies for this devastating disease.

Although little is known regarding the cause of ALS, accumulating evidence suggests that increased inflammatory responses and oxidative stress alongside glial cell dysfunction play crucial roles in disease pathogenesis; this is supported by clinical studies showing infiltration of immune cells and heightened inflammatory cytokine profiles in the central nervous system of ALS patients [7, 8]. Although cytokine data are inconsistent across studies, a meta-analysis by Chen et al. reported that granulocyte-colony stimulating factor, interleukin-2 (IL-2), IL-15, IL-17, monocyte chemotactic protein-1, macrophage inflammatory protein-1*α*, tumor necrosis factor-*α* (TNF-*α*), and vascular endothelial growth factor levels in the cerebrospinal fluid were significantly elevated in patients with ALS when compared with controls [9]. Another meta-analysis performed by our group including 25 studies clarified the peripheral blood inflammatory cytokine profile in ALS, which revealed elevated blood TNF-*α*, TNF receptor 1, IL-6, IL-1*β*, IL-8, and vascular endothelial growth factor levels in patients with ALS relative to control subjects [10]. In addition, a substantial number of clinical studies have analyzed oxidative stress markers in ALS and demonstrated that levels of prooxidative stress markers, malondialdehyde (MDA) and 8-hydroxyguanosine (8-OHdG) were increased in the peripheral blood of ALS patients [11, 12]. In contrast, decreased antioxidant glutathione and uric acid levels were observed in ALS patients [13, 14]. However, other studies have reported unaltered levels of antioxidants in patients with ALS compared to controls [15, 16]. Due to the heterogeneity of the clinical data on oxidative stress markers, the profile of oxidative stress markers in ALS patients remains unclear.

To better understand the etiology of ALS and potentially use oxidative stress markers for the diagnosis and prognosis of ALS patients, we reviewed PubMed and Web of Science systematically and pooled the data from the included studies to clarify the oxidative stress marker profile in patients with ALS.

## 2. Materials and Methods

This systematic review and meta-analysis was performed according to the instructions that are recommended by the PRISMA statement (Preferred Reporting Items for Systematic Reviews and Meta-Analysis) [17].

### 2.1. Literature Search

Two independent investigators manually reviewed English-language articles on PubMed and Web of Science from May 2018 to September 2018. The search terms for the systematic review were the following: (oxidative stress or catalase or hydroxyguanosine or malondialdehyde or uric acid or ceruloplasmin or glutathione or transferrin or low density lipoprotein or copper or cholesterol) and amyotrophic lateral sclerosis. We included original articles that reported peripheral blood levels of oxidative stress markers in ALS patients and control subjects.

### 2.2. Data Extraction

We extracted sample size, mean oxidative stress marker concentrations, *P* values, and standard deviation (s.d.) as the primary outcomes for this meta-analysis. We also extracted additional data on age, gender (proportion of males), publication year, sampling source, disease duration, and diagnosis of potential moderator analyses. The demographic and clinical variables of the included studies in this meta-analysis are presented in Supplementary Table (available here).

### 2.3. Statistical Analysis

The Comprehensive Meta-Analysis Version 2 software (Biostat, Englewood, NJ, USA) was used to pool the oxidative stress marker data on ALS patients. The sample size, mean oxidative stress marker concentration, and s.d. were primarily used to generate the effective size (ES). Sample size and *P* values were used to generate ES if oxidative stress marker concentration data were not available. An ES was calculated as the standardized mean difference in oxidative stress marker concentrations between cases and controls and then converted to Hedge's *g* which provides ES adjustment for sample size [18]. The 95% confidence interval (95% CI) was used to estimate the statistical significance of the pooled ES. We performed random-effects meta-analysis for this study because we estimated that the true ES would be affected by between-study and within-study variations [19]. We also performed sensitivity analysis by removing one study at a time to evaluate whether the statistical significance between cases and controls for oxidative stress marker concentrations was influenced by a single study.

The statistical difference of between-study heterogeneity was evaluated using the Cochran *Q* test [20], whereby statistical significance was set at *P* value < 0.1. The impact of between-study heterogeneity was evaluated by the *I* ^2^ index, and *I* ^2^ of 0.25, 0.50, and 0.75 suggested small, medium, and high levels of heterogeneity, respectively. We then used unrestricted maximum-likelihood random-effects metaregressions of ESs to analyze whether age, gender, or publication year had moderating effects on the outcomes of the meta-analysis. The publication bias for the included studies in this meta-analysis was determined by Egger's test [21], which assesses the degree of funnel plot asymmetry. The statistical significance of this meta-analysis was set at *P* value < 0.05 unless stated otherwise.

## 3. Results

**Figure 1:**
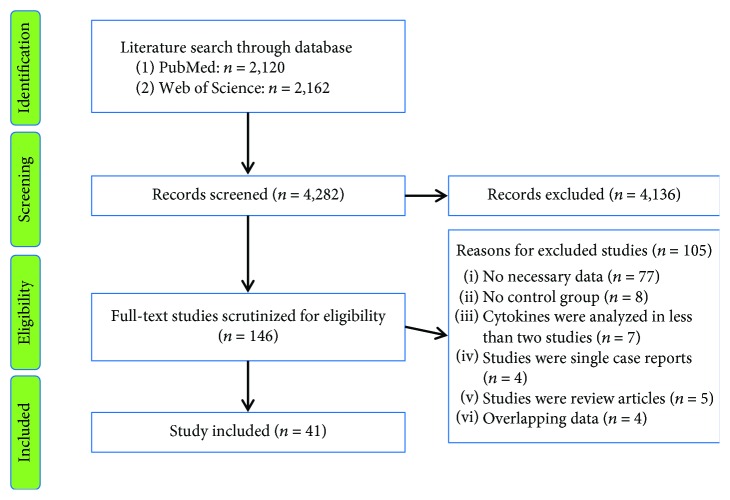
PRISMA flowchart of the literature search.

Our initial search with the keywords produced 2,120 records from PubMed and 2,162 records from Web of Science. After screening the titles and abstracts of the records, 146 articles were selected for full-text scrutiny. Of the 146 studies, 105 studies were excluded due to the following reasons: no necessary data (*n* = 77); without a control group (*n* = 8); oxidative stress markers were analyzed in less than two studies (*n* = 7); studies were single case reports (*n* = 4); studies were review articles (*n* = 5); or data overlapped with other studies (*n* = 4). A final total of 41 articles with 4,588 ALS patients and 6,344 controls were included in this meta-analysis [11–16, 22–56] (for the flowchart, see Figure 1).

### 3.1. Main Associations of Blood Oxidative Stress Markers with ALS

**Figure 2:**
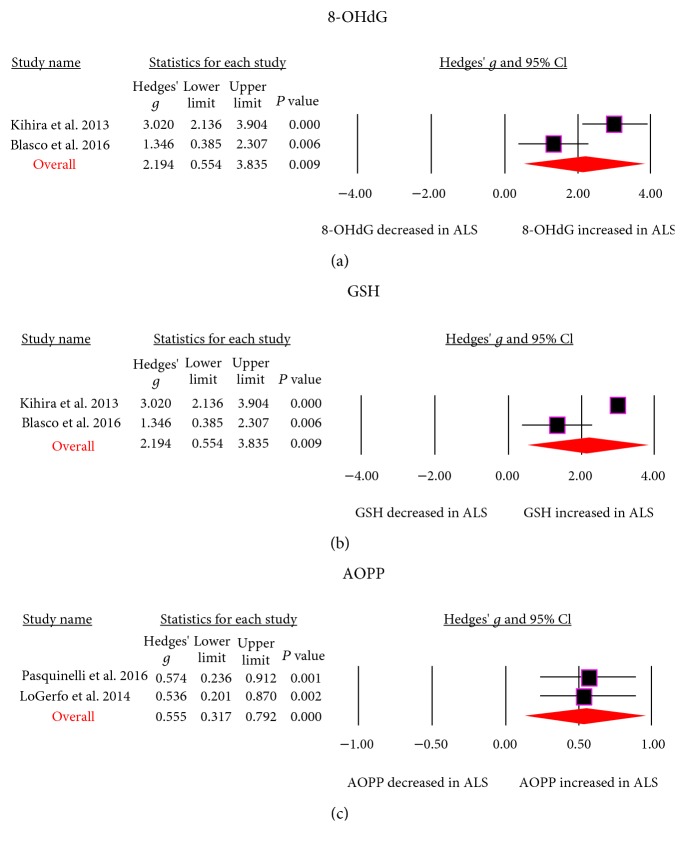
Studies of blood 8-OHdG, GSH, and AOPP in amyotrophic lateral sclerosis. Forest plot displaying random-effects meta-analysis results of the association between 8-OHdG (a), GSH (b), AOPP (c), and amyotrophic lateral sclerosis. GSH: glutathione; AOPP: Advanced Oxidation Protein Product; 8-OHdG: 8-hydroxyguanosine.

**Figure 3:**
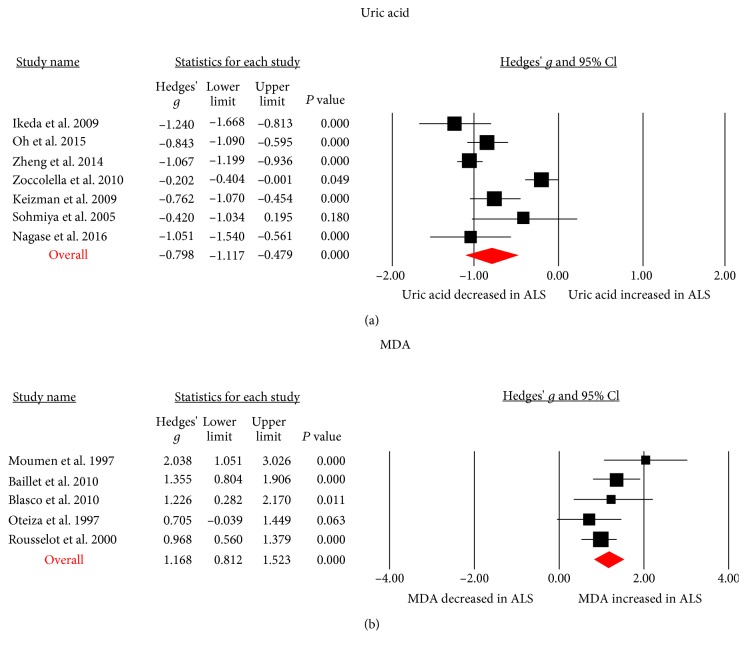
Studies of blood uric acid and MDA in amyotrophic lateral sclerosis. Forest plot displaying random-effects meta-analysis results of the association between uric acid (a), MDA (b), and amyotrophic lateral sclerosis. MDA: malondialdehyde.

Results from the meta-analysis showed that blood MDA (Hedges' *g*, 1.168; 95% CI, 0.812 to 1.523; *P* < 0.001), 8-OhDG (Hedges' *g*, 2.194; 95% CI, 0.554 to 3.835; *P* = 0.009), and Advanced Oxidation Protein Product (AOPP, Hedges' *g*, 0.555; 95% CI, 0.317 to 0.792; *P* < 0.001) levels were significantly elevated in patients with ALS when compared with controls, whereas blood uric acid (Hedges' *g*, -0.798; 95% CI, -1.117 to -0.479; *P* < 0.001) and glutathione (Hedges' *g*, -1.636; 95% CI, -3.020 to -0.252; *P* = 0.02) levels were significantly decreased in ALS patients (Figures 2 and 3 and Table 1). In addition, we did not observe significant differences between ALS patients and controls for blood Cu, superoxide dismutase (SOD), glutathione peroxidase, coenzyme-Q10 (Co-Q10), ceruloplasmin, total cholesterol, triglycerides, high-density lipoprotein (HDL), low-density lipoprotein (LDL), and transferrin levels (Table 1).

### 3.2. Investigation of Heterogeneity

For the fifteen oxidative stress markers analyzed in the meta-analysis, AOPP and ceruloplasmin did not show between-study heterogeneity. MDA showed small levels of between-study heterogeneity, Co-Q10 showed moderate levels of between-study heterogeneity, and high levels of heterogeneity among studies were found for the other eleven markers (Table 1).

We next explored whether potential moderators accounted for the heterogeneity for the ALS-associated five oxidative stress markers. Given that blood MDA and AOPP showed low levels of between-study heterogeneity, and due to the limited number of studies with small size analyzing 8-OHdG and glutathione levels, we performed metaregression and subgroup analyses on uric acid. As shown in the Supplementary Table, the information on disease duration of ALS patients was limited. We therefore conducted metaregression analyses according to age, gender, and publication year. Metaregression analyses suggested that publication year, gender, and age did not significantly affect the results of the meta-analysis (*P* > 0.05 in all analyses).

Next, we performed subgroup analyses based on the sampling source. Uric acid levels were reduced both in the serum (Hedges′ *g* = −0.810, 95%CI = −1.190 to − 0.431, *P* < 0.001) and the plasma (Hedges′ *g* = −0.764, 95%CI = −1.380 to − 0.148, *P* = 0.015) of ALS patients when compared with those of the controls. However, between-study heterogeneity was increased for the serum studies (*Q* = 53.542; df = 4; *I* ^2^ = 92.529; *P* < 0.001) but reduced for the plasma studies (*Q* = 2.478; df = 1; *I* ^2^ = 59.643; *P* = 0.115).

Furthermore, we performed sensitivity analysis and showed that the significant association between blood uric acid and ALS was not influenced by any individual study.

**Figure 4:**
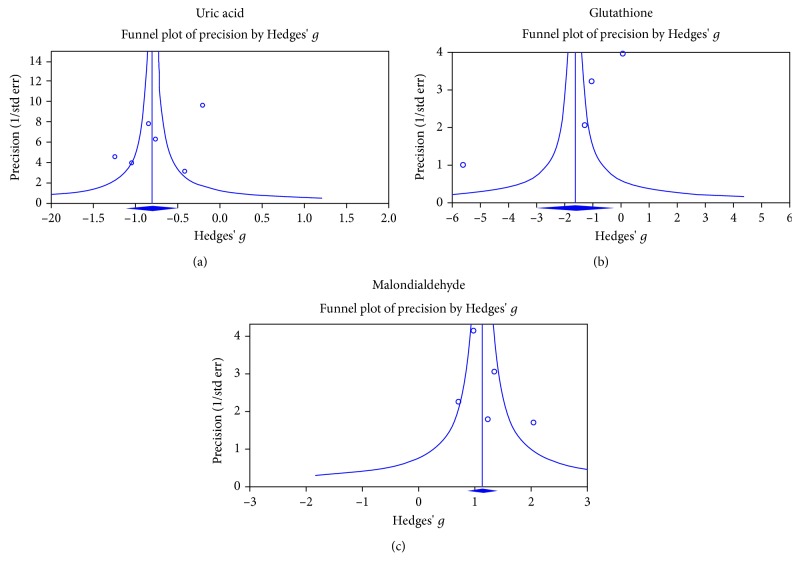
Funnel plots examining publication bias in studies comparing blood uric acid (a), GSH (b), and MDA (c) levels between cases and controls. GSH: glutathione; MDA: malondialdehyde.

Inspection of funnel plots visually indicated no publication bias for studies analyzing uric acid or MDA levels in ALS patients (Figure 4); these were confirmed by the Egger test (Table 1, *P* > 0.1). However, the funnel plot suggested there may be publication bias for studies analyzing glutathione levels in ALS patients (Figure 4). Further, the Egger test suggested a trend for publication bias for glutathione (Table 1, *P* = 0.061). To analyze the effect of potential publication bias, we used the classic fail-safe N^18^ to compute the number of missing studies (with mean effect of zero) that would require to bring the *P* value above 0.05 for glutathione, and the analysis showed that 30 studies would need to be added to generate a nonsignificant association between glutathione and ALS, suggesting that potential publication bias is unlikely to significantly affect the positive outcome of the present study. Due to the limited number of studies, we were unable to perform publication bias analysis for 8-OHdG and AOPP.

## 4. Discussion

This meta-analysis included 41 studies with 4,588 ALS patients and 6,344 control individuals analyzing 15 oxidative stress markers from the blood. The results suggest that MDA (the important end product for lipid peroxidation), 8-OhdG (a marker for DNA damage), and AOPP were significantly elevated in the blood of ALS patients when compared with control individuals. In addition, we found that the levels of antioxidant glutathione and uric acid were significantly downregulated in patients with ALS. However, other oxidative stress markers including Cu, SOD, glutathione peroxidase, ceruloplasmin, triglycerides, total cholesterol, LDL, HDL, Co-Q10, and transferrin were not significantly associated with ALS. For the five dysregulated oxidative stress markers in ALS patients, the results associated with ESs of 8-OHdG, MDA, and GSH were large, and the ESs were medium to large for AOPP and uric acid. Sensitivity analysis indicated that no individual study influenced the significantly decreased blood uric acid levels in ALS patients, and no publication bias risks were observed for studies analyzing uric acid and MDA concentrations as determined by funnel plots and the Egger test, indicating the robustness of the results from our present study.

Although our study is the first to use a meta-analytic technique to clarify the oxidative stress marker profile in patients with ALS, it is unclear whether oxidative stress has causal effect for ALS onset and/or development. However, the important role of oxidative stress in the pathogenesis of ALS is supported by the fact that the mutations in the gene encoding the cytosolic antioxidant enzyme-SOD1 cause ALS [57]. In addition, mutant SOD1 transgenic mice exhibited age-dependent motor neuron degeneration accompanied by the biochemical changes in the nerve cells [58]. Moreover, it has been reported that uric acid levels were negatively correlated with the disease progression in ALS patients [44]. Collectively, these previous findings and our pooled clinical data of the dysfunction between the oxidation and antioxidant systems in ALS patients support the hypothesis that oxidative stress is central in the pathogenesis of ALS.

Our analyses further showed that most of the oxidative stress markers had high levels of heterogeneity among studies. However, for the five oxidative stress markers that were dysregulated in the patients with ALS, AOPP did not show between-study heterogeneity and MDA showed small levels of heterogeneity, suggesting the reproducibility of these results. In addition, we conducted subgroup and metaregression analyses to address moderators that may explain heterogeneity for uric acid. The results indicated that gender, age, and publication year did not contribute to the between-study heterogeneity. Although subgroup analyses based on sampling source revealed that between-study heterogeneity was reduced in plasma studies analyzing uric acid levels, the lower heterogeneity is likely due to the low power of the test for heterogeneity used in meta-analyses with smaller numbers of studies. Other clinical variables including medication status and disease duration may also contribute to between-study heterogeneity. However, most of the studies included in the meta-analysis did not provide this information, thus preventing us from performing subgroup or metaregression analyses to assess whether these factors contributed to between-study heterogeneity. Indeed, a study reported that lithium and valproate cotreatment increased the survival of patients with ALS and the treatment also significantly increased blood glutathione levels in these patients [24].

The second limitation of this study is how much the alterations in oxidative stress markers in the peripheral blood reflect changes in the central nervous system. However, Djordjevic et al. reported that patients with ALS had significantly increased cerebrospinal fluid AOPP levels relative to control subjects [59]. Moreover, Murata et al. demonstrated that ALS patients had higher CSF 8-OHdG concentrations than control subjects [60]. These results support the “peripheral as a window to the brain” hypothesis. Further studies are necessary to translate these findings into practical clinical use. The third limitation of this study is that some oxidative stress markers analyzed in this study such as glutathione peroxidase had a limited number of studies with small sample sizes; therefore, it is difficult to determine significant associations between these markers and ALS. In addition, several other important oxidative stress markers such as catalase were not analyzed in the meta-analysis due to the lack of clinical studies on these markers. Future studies should clarify the role of oxidative stress in the onset and development of ALS.

In addition to prooxidative stress imbalance observed in ALS, a large number of studies have measured oxidative stress markers in other neurodegenerative diseases including Alzheimer's disease [61] and Parkinson's disease [62]. Due to the heterogeneous etiologies of these diseases, it is not surprising that results are inconsistent across studies comparing oxidative stress marker levels between patients with Alzheimer's disease or Parkinson's disease and controls. To address the inconsistent clinical data, Schrag et al. performed a meta-analysis and reported that patients with Alzheimer's disease were accompanied by reduced uric acid levels and increased MDA levels in the peripheral blood [63]. In addition, a systematic review and meta-analysis performed by Wei et al. showed that blood 8-OHdG and MDA levels were elevated in Parkinson's disease patients, whereas uric acid and glutathione levels were downregulated in these patients [64]. The dysregulated profiles of oxidative stress markers in Parkinson's disease and Alzheimer's disease were similar to the findings from our meta-analysis on profiles of oxidative stress markers in ALS, suggesting a common pathway that confers vulnerability to the development of these neurodegenerative diseases.

In conclusion, the findings from the present study revealed increased 8-OhdG, MDA, and AOPP levels and reduced uric acid and glutathione levels in the peripheral blood of ALS patients. Our results clarify the oxidative stress marker profile in the blood of ALS patients and strengthens the clinical evidence that prooxidative imbalances contribute to ALS pathophysiology.

## Figures and Tables

**Table 1:** Summary of comparative outcomes for measurements of blood oxidative stress marker levels.

Cytokine	No. of studies	No. with ALS/controls	Main effect			Heterogeneity				Publication bias	
			Hedges' *g* (95% CI)	*z* score	*P* value	*Q* statistic	df	*P* value	*I* ^2^ statistic	Egger intercept	*P* value
Uric acid	7	961/1185	-0.798 (-1.117 to -0.479)	-4.906	<0.001	56.034	6	<0.001	89.292	0.910	0.750
8-OHdG	2	18/61	2.194 (0.554 to 3.835)	2.622	0.009	6.315	1	0.012	84.165	NA	NA
MDA	5	123/120	1.168 (0.812 to 1.523)	6.441	<0.001	5.793	4	0.215	30.950	1.61721	0.38172
GSH	4	71/78	-1.636 (-3.020 to -0.252)	-2.318	0.020	36.112	3	<0.001	91.692	-7.014	0.061
AOPP	2	147/133	0.555 (0.317 to 0.792)	4.571	<0.001	0.025	1	0.875	0.000	NA	NA
Ceruloplasmin	3	46/46	-0.052 (-0.475 to 0.372)	-0.239	0.811	2.168	2	0.338	7.742	2.538	0.403
Cu	10	361/471	0.014 (-0.337 to 0.366)	0.080	0.937	39.670	9	<0.001	77.313	0.09686	0.94797
Glutathione peroxidase	4	91/110	-0.679 (-1.732 to 0.373)	-1.265	0.206	29.687	3	<0.001	89.895	-4.92304	0.30857
Total cholesterol	13	2161/3870	0.144 (-0.095 to 0.383)	1.184	0.236	160.185	12	<0.001	92.509	1.50578	0.44161
HDL	9	2024/3722	-0.198 (-0.625 to 0.228)	-0.912	0.362	345.128	8	<0.001	97.682	2.99958	0.54140
LDL	9	2024/3722	0.121 (-0.283 to 0.524)	0.587	0.557	307.948	8	<0.001	97.402	2.74193	0.55440
SOD	5	96/184	-0.203 (-0.904 to 0.497)	-0.569	0.569	22.703	4	<0.001	82.381	9.11205	0.01197
Co-Q10	4	95/128	0.040 (-0.468 to 0.548)	0.156	0.876	9.953	3	0.019	69.858	-2.10679	0.75503
Transferrin	5	954/592	0.244 (-0.219 to 0.707)	1.032	0.302	50.330	4	<0.001	92.052	5.24433	0.09393
Triglyceride	8	1743/1633	-0.154 (-0.622 to 0.314)	-0.645	0.519	265.560	7	<0.001	97.364	4.85477	0.32384

Abbreviations: df: degrees of freedom; ALS: amyotrophic lateral sclerosis; 8-OHdG: 8-hydroxyguanosine; MDA: malondialdehyde; SOD: superoxide dismutase; AOPP: Advanced Oxidation Protein Product; LDL: low-density lipoprotein; HDL: high-density lipoprotein; Co-Q10: coenzyme-Q10; NA: not available.

## Data Availability

The data used to support the findings of this study are included within the article.

## Supplementary Materials

Supplementary Materials Supplementary Table: characteristics of included studies measuring peripheral blood oxidative stress marker levels.
