# Supplementary material for: Aberrations in Oxidative Stress Markers in Amyotrophic Lateral Sclerosis: A Systematic Review and Meta-Analysis
Source: Oxid Med Cell Longev. 2019 Jun 9;2019:1712323. doi: 10.1155/2019/1712323 (PMC6590548; doi:10.1155/2019/1712323)
Supplement: Supplementary Materials — Supplementary Table: characteristics of included studies measuring peripheral blood oxidative stress marker levels. [file 1712323.f1.pdf]

Supplementary Table: Characteristics of included studies measuring peripheral blood oxidative stress marker levels

Author/year	Country	groups	N (ALS/ Control)	Gender (% Male) (ALS/ Control)	Age (ALS/Control)	Disease Duration (months)	Diagnosis	Oxidative Stress Marker Measured	Sample Origin
Ahmed et al. 2018	Australia	ALS/ NC	37/32	70/38	55.9/64.7	20.4	NA	Total cholesterol, LDL, HDL,TG	Blood
Baillet et al. 2010	France	ALS/ NC	31/30	55/37	63/39.4	NA	El Escorial	MDA, GSH, Cu	Plasma
Barros et al. 2018	Brazil	ALS/ NC	27/26	48/42	56/55.4	46	NA	Cu, Ceruloplasmin, Total cholesterol, LDL,HDL,TG	Plasma &Serum
Benedetti et al. 2017	Italy	ALS/ NC	6/5	50/NA	67.2/63.7	87.6	El Escorial	Cu	Blood
Blasco et al. 2016	France	ALS/ NC	9/10	44.4/40	65.7/65.4	NA	El Escorial	SOD, GSH-Px, GSH, 8-OHdG, MDA, Cu	Blood, Serum &Plasma
Boll et al. 2014	México	ALS/ NC	18/31	80/50	56.06/52.08	46.55	El Escorial	GSH-Px, GSH, SOD	Plasma
Chio et al. 2009	Italy	ALS/ NC	658/658	52/52	NA	NA	El Escorial	Total cholesterol, HDL, LDL,TG	Serum
Conti et al. 2008	Italy	ALS/ NC	5/6	NA	NA	NA	El Escorial	Ceruloplasmin	Plasma
Delaye J.B. et al. 2017	France	ALS/ NC	30/29	50/51.8	66.5/66.3	NA	NA	Lipoprotein, Total cholesterol, LDL ,HDL	Serum
Dupuis et al. 2008	France	ALS/ NC	369/286	57/NA	57.5/NA	NA	El Escorial	Total cholesterol, HDL, LDL,TG	Serum
Ehrhart et al. 2015	USA	ALS/ NC	13/7	92/43	53.85 /57.71	41.77	El Escorial	GSH	Serum
Huang et al. 2015	China	ALS/ NC	413/400	NA	NA	21.8	El Escorial	Total cholesterol, HDL,LDL,TG	Serum
Ihara et al. 2005	Japan	ALS/ NC	28/16	NA/NA	61.24/63.25	NA	NA	SOD, Cu	Plasma
Ikeda et al. 2009	Japan	ALS/ NC	49/50	65/60	58.8/59.1	NA	El Escorial	Uric acid	Serum
Ikeda et al. 2012	Japan	ALS/ NC	92/92	62/62	58.8/59.2	23.7	El Escorial	transferrin, HDL, LDL, TG, Total cholesterol	Serum
Kanisa et al. 1997	Greece	ALS/ NC	28/59	68/61	57.9/43.8	NA	NA	Cu	Serum
Keizman et al. 2009	Israel	ALS/ NC	86/86	65/65	58/57.8	NA	El Escorial	Uric acid	Serum
Kihira et al. 2013	Japan	ALS/ NC	9/51	67/35	65.3/73	NA	El Escorial	Cu, 8-OHdG	Serum
Kim et al. 2017	Korea	ALS/ NC	39/33	49/48	53.6/50.52	NA	El Escorial	Total cholesterol	Serum
LoGerfo et al. 2014	Italy	ALS/ NC	73/68	49/65	63.3/69.3	NA	NA	AOPP	Plasma
Mitchell et al. 2010	USA	ALS/ NC	10/12	NA	NA	NA	El Escorial	Transferrin	Plasma
Mokuno et al. 1996	Japan	ALS/ NC	20/120	NA	NA	NA	NA	SOD	Serum
Molina et al. 2000	Spain	ALS/ NC	30/42	47/56	61.8/59.7	24	El Escorial	Co-Q10	Serum
Moumen et al. 1997	France	ALS/ NC	21/7	24/72	NA	NA	El Escorial	GSH-Px, SOD, MDA	Plasma

Nadjar et al. 2012	France	ALS/ NC	694/297	52/58	61.85 /48.99	NA	NA	Transferrin	Serum
Nagase et al. 2016	Japan	ALS/ NC	26/55	54/69	60.9/60.1	NA	NA	Total cholesterol, Uric acid, Co-Q10	Plasma
Oh et al. 2015	Korea	ALS/ NC	136/136	51/51	55.0/54.7	13.6	El Escorial	Uric acid	Serum
Oteiza et al. 1997	Argentina	ALS/ NC	19/11	46/73	NA	NA	NA	Co-Q10	Plasma
Pasquinelli et al. 2016	Italy	ALS/ NC	74/65	NA	62.4/74	83.2	NA	AOPP	Plasma
Peters et al. 2016	USA	ALS/ NC	163/229	98/94	62.9/63.4	NA	El Escorial	Cu	Blood
RadunoviC et al. 1997	UK	ALS/ NC	46/31	59/45	55.4/56.8	NA	NA	Cu	Plasma &Serum
Rousselot et al. 2000	France	ALS/ NC	43/62	60/47	60/60	33.6	El Escorial	GSH-Px	Plasma
Sohmiya et al. 2005	Japan	ALS/ NC	20/20	65/65	61.2/62.1	46.9	El Escorial	Uric acid, Co-Q10,Total cholesterol	Plasma
Sutedja et al. 2010	Netherlands	ALS/ NC	303/2100	57/54	64/59	NA	El Escorial	Total cholesterol, LDL, HDL	Plasma
To´rsdo´ttir et al. 2000	Iceland	ALS/ NC	14/14	50/50	61.7/61.7	61.2	El Escorial	Cu, Ceruloplasmin	Plasma &Serum
Veyrat-Durebex et al. 2014	France	ALS/ NC	104/145	52/53	67.6/68.4	NA	El Escorial	Transferrin	Serum
Wuolikainen et al. 2014	Sweden	ALS/ NC	52/40	56/53	58.89/61.6	NA	EFNS consensus criteria	Total cholesterol, TG	Plasma
Yang et al. 2013	Korea	ALS/ NC	95/99	63/65	54.14/52.52	NA	El Escorial	Total cholesterol, LDL, HDL, TG	Serum
Zheng et al. 2014	China	ALS/ NC	512/501	58/58	53.3/53.4	38.4	El Escorial	Uric acid	Serum
Zheng et al. 2017	China	ALS/ NC	54/46	61/	53.85/51.26	14.99	El Escorial	Transferrin	Serum
Zoccolella et al. 2010	Italy	ALS/ NC	132/337	61/65	65.7/64	21	El Escorial	Uric acid	Serum

Abbreviations: ALS, amyotrophic lateral sclerosis; NC, normal control; 8-OHdG, 8 hydroxyguanosine; MDA, Malondialdehyde; SOD, superoxide dismutase; AOPP, Advanced Oxidation Protein Product; LDL, low density lipoprotein; HDL, high density lipoprotein; Co-Q10, coenzyme-Q10; TG, Triglycerides; AOPP, advanced oxidation protein products; GSH-Px, glutathione peroxidase; NA, not available.
